# Gold nanoparticles stabilize peptide-drug-conjugates for sustained targeted drug delivery to cancer cells

**DOI:** 10.1186/s12951-018-0362-1

**Published:** 2018-03-30

**Authors:** Kalishwaralal Kalimuthu, Bat-Chen Lubin, Andrii Bazylevich, Gary Gellerman, Ofer Shpilberg, Galia Luboshits, Michael A. Firer

**Affiliations:** 1 Dept. Chemical Engineering, Ariel University, 40700 Ariel, Israel; 2 Eastern R&D Center, Ariel, 40700 Israel; 3 Dept. Chemical Sciences, Ariel University, 40700 Ariel, Israel; 4 Tel Aviv & Pre-Med Faculty, Assuta Medical Center, Institute of Hematology, Ariel University, 40700 Ariel, Israel; 5 Ariel Center for Applied Cancer Research, Ariel University, 40700 Ariel, Israel

**Keywords:** Targeted drug delivery, Peptide drug conjugates, Phage display, Gold nanoparticles

## Abstract

**Background:**

Peptide-drug-conjugates (PDCs) are being developed as an effective strategy to specifically deliver cytotoxic drugs to cancer cells. However one of the challenges to their successful application is the relatively low stability of peptides in the blood, liver and kidneys. Since AuNPs seem to have a longer plasma half-life than PDCs, one approach to overcoming this problem would be to conjugate the PDCs to gold nanoparticles (AuNPs), as these have demonstrated favorable physico-chemical and safety properties for drug delivery systems. We set out to test whether PEG coated-AuNPs could provide a suitable platform for the non-covalent loading of pre-formed PDCs and whether this modification would affect the bioavailability of the PDCs and their cytotoxicity toward target cancer cells.

**Methods:**

Peptides specifically internalized by A20 murine lymphoma cells were isolated from a phage library displaying 7mer linear peptides. Peptide specificity was validated by flow cytometry and confocal microscopy. PDCs were synthesized containing a selected peptide (P4) and either chlorambucil (Chlor), melphalan (Melph) or bendamustine (Bend). Gold nanoparticles were sequentially coated with citrate, PEG-6000 and then PDC (PDC-PEG-AuNP). The physico-chemical properties of the coated particles were analyzed by electrophoresis, TEM, UV–VIS and FTIR. Stability of free and PDC-coated AuNP was determined.

**Results:**

Biopanning of the phage library resulted in discovery of several novel peptides that internalized into A20 cells. One of these (P4) was used to synthesize PDCs containing either Chlor, Melph or Bend. All three PDCs specifically killed A20 target cells, however they had short half-lives ranging from 10.6 to 15.4 min. When coated to PEG-AuNPs, the half-lives were extended to 21.0–22.3 h. The PDC-PEG-AuNPs retained cytotoxicity towards the target cells. Moreover, whereas pre-incubation for 24 h of free PDCs almost completely abolished their cytotoxic activity, the PDC-PEG-AuNPs were still active even after 72 h pre-incubation.

**Conclusions:**

Peptide-drug-conjugates hold potential for improving the target efficacy of chemotherapeutic drugs, however their short half-lives may limit their application. This hurdle can be overcome by easily conjugating them to gold nanoparticles. This conjugation also opens up the possibility of developing slow release formulations of targeted drug delivery systems containing PDCs.

**Electronic supplementary material:**

The online version of this article (10.1186/s12951-018-0362-1) contains supplementary material, which is available to authorized users.

## Background

Efficient systems for the targeted delivery of drugs into cancer cells have not yet reached the clinic. These systems are important to develop because their potential to minimize off-target side effects may lead to a significant widening of the therapeutic window. However to achieve this goal it will in part, be necessary to improve the stability of the carrier-drug constructs so as to ensure restricted release of the drug payload to the tumor cell or at least its local environment.

The strategy of targeted drug delivery (TDD) to tumor cells is based on cell surface heterogeneity between normal cells and cancer cells. For example the targeting of over expressed receptors by high affinity biomolecular carriers such as antibodies can mitigate the selectivity problems of chemotherapy [1]. However, despite the accelerated clinical development of antibody–drug-conjugates, there remain a number of challenges to their efficient use [1, 2]. Our research focus is to use much smaller carriers such as peptides and we have demonstrated that such peptide-drug-conjugates (PDCs) are not only specific for their target cells, but they do not induce outgrowth of drug resistant cells, can reverse drug resistance and can deliver multi-drug payloads [3–8].

One of the remaining challenges to the successful application of PDCs for cancer therapy is the relatively low stability of peptides to enzymatic hydrolysis in the blood, liver and kidneys [9]. One approach to overcoming this problem would be to conjugate the PDC to nanoparticles, as these have demonstrated improved pharmacokinetic and pharmacodynamics properties when used as drug delivery systems [10, 11]. Indeed gold nanoparticles (AuNPs), with their favorable physico-chemical and safety properties [12, 13] and ease of synthesis [14] might be appropriate agents for enhanced PDC delivery. Similar to organic colloids, ligand modification is not necessary for chemi-adsorption on to the colloidal gold surface, so AuNPs can easily be conjugated to different molecules such as peptides, enzymes, DNA and small molecule drugs [15] and can lead to improved drug stability and reduced side effects [16, 17]. Unfortunately, despite the huge amount of research being conducted in applying nanoparticles to cancer therapy (7174 Web of Science entries for 2016 alone), a recent 10-year literature analysis by Wilhelm et al. showed that overall, only about 0.7% of the administered dose of nanoparticles actually reach a solid tumor [18, 19].

Since AuNPs seem to have a longer plasma half-life than PDCs, we thought to use them not as a drug delivery mechanism but rather as a PDC stabilizer. We hypothesized that PDCs conjugated to AuNPs may improve the former’s bioavailability. We set out to test whether PEG coated-AuNPs could provide a suitable platform for the non-covalent loading of pre-formed PDCs and whether this modification would affect the bioavailability of the PDCs and their cytotoxicity toward target cancer cells.

## Methods

### Cell culture

The primary normal cells HMVEC-dBlAd-Adult dermal blood microvascular (CC-2811) and human renal proximal tubule cells—(CC2553) were purchased from Lonza (Switzerland) and grown in EGM 2MV bullet kit-(cc4147), and REGM bullet kit-(cc3191) media respectively, as recommended by the supplier. 3T3 mouse fibroblasts, human HL-60, NB4 and murine A20 leukemic cells (purchased from ATCC) were grown in RPMI 1640, 10% FBS, antibiotics and 2 mM l-glutamine (Biological Industries, Bet Haemek, Israel). Mouse MOPC 315.BM cells (kindly provided by Prof. Bjarne Bogen; University of Osla, Norway) were grown in RPMI 1640 GlutaMAX (Gibco) supplemented with 1% MEM NEAA 100× (Gibco), 1% sodium pyruvate (Gibco), 0.005% 1 M I-thioglycerol (Sigma), 0.03% Gensumycin 40 mg/ml (Sanofi Aventis) and 10% Fetal Bovine Serum (Gibco). Cells were cultured at 37 °C in 5% CO_2_ and passaged at 3- to 4-day intervals at sub-confluency. Murine cell suspensions of heart and kidney were prepared from Balb/c mice by mincing the tissue into PBS. The suspension was passed through a 70 µm mess and the cells washed thrice in PBS.

### Removal of irrelevant phage clones by in vitro negative selection

To remove non-relevant phage clones before exposure to the target cells, a linear heptapeptide phage display library (Ph.D.-7L New England Biolabs, MA, USA) was sequentially exposed to a series of normal, control cells in the following order: HUVEC, 3T3, murine heart cell suspension, murine kidney cell suspension, human peripheral blood mononuclear cells, human erythrocytes.

In each case, 1 × 10^6^ log-phase cells were washed gently with PBS and re-suspended in 1 ml of RPMI. 10 microliters of phage particles (2 × 10^11^) were added 1 ml of cell suspension and the mixture was incubated for 1 h at 37 °C with gentle shaking. The mixture was then centrifuged at 1500 rpm and the supernatant containing the unbounded phage was collected. A small portion (3–5 µl) was retained and titrated to determine the decrease in phage titer resulting from each absorption. The remainder was used for the next stage of negative panning. The library was similarly exposed to matrigel. After the final absorption, the resultant negatively selected phage pool was termed “adsorbed phage library”.

### Positive selection for A20 internalized phage peptides

A20 cells (1 × 10^6^) were incubated with the adsorbed phage library for 1 h at 37 °C with gentle shaking. The mixture was then centrifuged for 5 min 14,000 rpm, the supernatant was discarded and the cells washed with PBS. They were then incubated for 10 min with 200 µl of 0.1 M HCl (pH 2.2) to elute cell surface-bound phage. This recovered solution was immediately neutralized by addition of 30 µl 0.5 M Tris (pH 9). The cells were then lysed for 1 h on ice in 200 µl of 30 mMTris–HCl and 1 mM EDTA (pH 8.0) to recover the cell-internalized phage fraction. After titration of this lysate, 10–15 isolated plaques were individually recovered, amplified and their DNA extracted according the library manufacturer’s instructions. Peptide inserts were sequenced by Sanger sequencing (Hylabs, Israel) using primers supplied by New England Biolabs and peptide amino acid sequences were deduced.

### Peptide synthesis

Peptides were synthesized using solid phase synthesis either as free peptides or conjugated at the *N*-terminus to FITC (China Peptides Co., Ltd, Shanghai, China). The products were purified (> 95%) by HPLC and characterized by mass spectrometry.

### Specificity of peptide binding

To assess the binding capacity of the candidate peptides, peptide-FITC conjugates were incubated at 0, 4 or 8 µM with 10^6^ A20 cells at 37 °C for 30 min. Following two washings with PBS, the cells were re-suspended in 200 μl of PBS and analyzed for peptide binding on a Becton Dickson FACS Calibur cell analyzer equipped with an argon ion laser (15 W) at 488 nm with a 530/30 DF filter. For each sample ~ 10^4^ cells were processed. FlowJo software was used to analyze the collected data. Peptides demonstrating the strongest binding were then tested for binding specificity by exposure (using the same conditions as above) to the following cells: mouse peripheral mononuclear cells (PBMC), murine MOPC 315.BM and the human leukemic cell lines (HL-60 and NB-4).

### Confocal microscopy

A20 cells (10^5^ cells/well) were plated into 8 chamber glass slides and then incubated with FITC—peptide conjugates (8 µM). After 30 min of incubation, intracellular distribution of the fluorescence was analyzed using a Zeiss confocal microscope (LSM 510 Meta) equipped with a NA 1.4 × 63 oil-immersion Plan-Apochromat objective lens. After counterstaining of cells with DAPI, fluorescent images of tissue slides were acquired.

### Synthesis of peptide–drug conjugates (PDC)

Peptides P-4 and P-6 bound to 2-chlorotrityl resin (Additional file 1: Figure S1) were supplied by PepMic Co., Ltd, China. Drugs (Chlorambucil, Melphalan and Bendamustine) and other chemicals and solvents were purchased from Sigma-Aldrich (Rehovot, Israel). N-Boc melphalan was prepared by the method described by Xu et al. [20] and conjugated to the peptide via standard Fmoc-SPPS procedures. The solid phase based synthesis of peptide- Chlorambucil and Bendamustine PDC was performed by following a previously described procedure [21] The peptide-drug-conjugate were cleaved from resin using 95% TFA 5% DCM mixture, purified on preparative HPLC and dried by lyophilization.

LC/MS analyses were performed on an Agilent Technologies 1260 Infinity (LC) 6120 quadruple (MS) with an Agilent SB-C18 column, 2.1 × 50 mm, column temperature 50 °C, eluent water-acetonitrile (ACN) that contained 0.1% of formic acid. HPLC purifications were carried out on an ECOM preparative system, with dual UV detection at 254 and 230 nm. Phenomenex Gemini^®^ 10 µm RP18 110 Å, LC 250 × 21.2 mm column was used. The column was kept at 25 °C. Eluent A (0.1% TFA in water) and B (0.1% TFA in ACN) were used. A typical elution was a gradient from 100% A to 100% B over 35 min at a flow rate of 25 ml/min.

### Synthesis of citrate-coated gold nanoparticles

Briefly, 5 ml of 60 mM Gold Chloride (Sigma) was added to 470 ml of deionized water and heated to boiling while being stirred. Upon boiling, 25 ml of 18 mM trisodium citrate was added to the solution and the reaction was terminated once a deep red solution color was obtained. The solution was allowed to cool to room temperature and the gold nanoparticles (AuNP) separated by centrifugation (15,000 rpm, 5 min). They were stored room temperature.

### PEG-6000 coated gold nanoparticles

1 g of methyl-PEG-6000 was added to a 10 ml solution of citrate-capped Au nanoparticles (50 mg) and stirred for 1 h allowing citrate to exchange with mPEG-6000, resulting in non-covalent attachment of PEG to the Au nanoparticles. Excess mPEG-6000 was removed by centrifugation at 15,000 rpm for 30 min and discarding the supernatant. The pellet was re-suspended in 1 M HEPES and stored in 4 °C.

### Determination of loading efficiency of PDC on PEG AuNPs

A 4 mM (3.9 mg) solution of each PDC was added to a1 ml PEG-Au NP solution and the mixture stirred for 30 min at room temperature. PDC loading efficiency was determined by centrifuging the mixture at 15,000 rpm for 5 min and determining the concentration of free PDC in the supernatant by LC/MS. No traces of PDC were found in the supernatant.

### Characterization of the PDC-PEG-AuNPs

#### Electrophoretic mobility assay

To obtain a qualitative measurement of PDC attachment to the NPs, gel electrophoresis of naked, PEG-coated and PDC-PEG-coated AuNPs was performed in a 1.5% agarose gel in TAE running buffer. The field strength was held constant at 120 V and the current was 97 mA. 50 μl of AuNP solutions were mixed with 50 µl of 1:9 glycerol/buffer solution and loaded onto the gel.

#### Morphology

The surface plasmon characteristics of the AuNP before and after coating were analyzed using a Varion Cary 50 Bio UV–VIS spectrophotometer. The morphology of the nanoparticle was determined by transmission electron microscopy (TEM). The nanoparticles were negatively stained with 2% phosphotungstic acid and placed on a carbon-coated copper grid. The particles were then examined with a JOEL JEM 1400 transmission electron microscope operating under an accelerating voltage of 80 kV. The micrographs of the nanoparticles were obtained using Olympus Keen view CCD camera attached to the microscope. FTIR analysis (Bruker Vertex 70) was used to characterize the chemical binding of both PEG and peptide drug conjugate to the surface of the nanoparticle.

### Stability of PDCs and PDC-coated PEG-Gold nanoparticles

Stock solutions were prepared by dissolving 5 mg of P4-chlorambucil, P4-melphalan or P4-bendamustine coated PEG-AuNPs in 1 mL of deionized water.

#### Chemostability

100 μl of stock solution were diluted to 1 ml of 0.01 M phosphate buffer solution pH 7.2 and incubated at 37 °C. At designated time points an aliquot was removed and centrifuged at 5000 rpm for 5 min. The supernatant was recovered, filtered through a 0.22 µM syringe and analyzed by LCMS for the presence of free drug. Results for each time point were expressed as mean % PDC integrity ± standard error, calculated from two independent experiments.

#### Biostability in liver homogenate

Mice were sacrificed by CO_2_ euthanasia. Livers were removed, and rinsed in cold 0.01 M PBS buffer pH 7.4. They were transferred to 10 ml Tris-HCI, pH 7.4 and homogenized in in a Potter–Elvehjem glass tissue grinder for 4 min. The tissue homogenate was centrifuged for 20 min at 4 °C, 14,000 rpm, and the supernatant was collected. Protein concentration was measured using the BCA protein assay using bovine serum albumin as a standard. The homogenate was used immediately or else stored in liquid nitrogen.

100 μl of each sample was added to 1 ml of liver homogenate (equivalent to 6 mg total protein). The samples were incubated at 37 °C and aliquots were taken during the different incubation period at different time intervals. The collected aliquots were immediately filtered, and analyzed by LC–MS.

### Cell cytotoxicity assay

To measure drug cytotoxic effect, cells were seeded into 96-wells Nunclon culture plates at 2.5 × 10^3^ cells per well and grown overnight at 37 °C. The spent medium was removed and the cells cultured for 72 h in fresh medium containing either free drug, PDCs, PEG-AuNPs or PDC-PEG-AuNPs at concentrations ranging from 0 to 50 µM (drug) filtered through a 0.22 µm syringe. Cellular metabolic status, used as a measure of cell viability, was then determined by replacing the culture medium with fresh medium containing XTT reagent (50 µl/well) and the cells were re-cultured for 2 h sat 37 °C. Absorbance in the wells was measured at 450 nm and subtracted from the reference absorbance at 630 nm. Culture medium was used as background control. All experiments were performed in triplicate on three separate occasions. Data are presented as mean ± S.D.

To further test the effect of nanoparticles conjugation on PDC activity, free drugs, PDCs and PDC-PEG-AuNPs were pre-incubated in growth medium for 24, 48 or 72 h at 37 °C after which time the solutions were added to fresh A20 cells. The cells were grown for a further 72 h and cytotoxicity was determined.

### Statistical analyses

Stability and cellular metabolism assays were performed in triplicate. Final results were calculated as the mean ± standard deviation of repeat experiments. Differences between groups were evaluated for statistical significance using the one-tailed *t* test for groups with equal variance. A p value of ≤ 0.05 was taken as statistically significant.

## Results

### Identification of phage peptides specifically internalized by A20 cells

Before exposure to the target cells, the stock Ph.D-7 linear phage display library was sequentially absorbed in vitro on a series of normal human and mouse cells and on Matrigel, in an effort to remove as many phage clones as possible that display peptides against normal cell surface and matrix polymer components. As shown in Additional file 1: Figure S2, this process reduced the stock concentration from ~ 3 × 10^10^ pfu/µl to ~ 10^6^ pfu/µl. This absorbed library was then amplified to expand the number of each of the remaining clones and to restore the initial phage concentration.

The absorbed library was then exposed to A20 cells. Unbound phage were removed and cell bound phage eluted. The cells were then lysed and internalized phage recovered and amplified. These phage were similarly subjected to two more exposure cycles on fresh A20 cells. Internalized phage from cycle 3 were titrated on bacterial lawns and 15 isolated plaques were randomly selected and designated P1, P2, P3…P15. ssDNA was extracted separately from each phage colony, the DNA sequences of the PIII displayed peptides from each colony obtained by Sanger sequencing and their corresponding peptide sequences derived. Table 1 shows the amino acid sequences of these peptides. Several colonies displayed the same peptide sequence indicating they were derived from the same clones as would be expected after three rounds of selection. From these results three clones, P-4, P-6 and P-8 were chosen for further study. Initial biochemical analysis of these sequences (http://protcalc.sourceforge.net/cgi-bin/protcalc) indicated that at physiological pH, P4 would be essentially not charged, while P6 and P8 would be negatively charged (− 2.2 and − 1.2 respectively).

**Table 1:** Peptide sequences of phage internalized by A20 cells and the frequency amongst the sequenced clones

Clone designation	Peptide sequence	Number of repeats
P-1	IIE GLY GLY ASN LEU SER ALA	1
P-2	GLY VAL ALA IIE THR MET LYS	2
P-4	HIS SER THR PRO SER SER PRO	7
P-6	ASN ASP LEU MET ASN ARG ALA	2
P-8	ASP SER SER LEU PHE ALA LEU	3

**Fig. 1:**
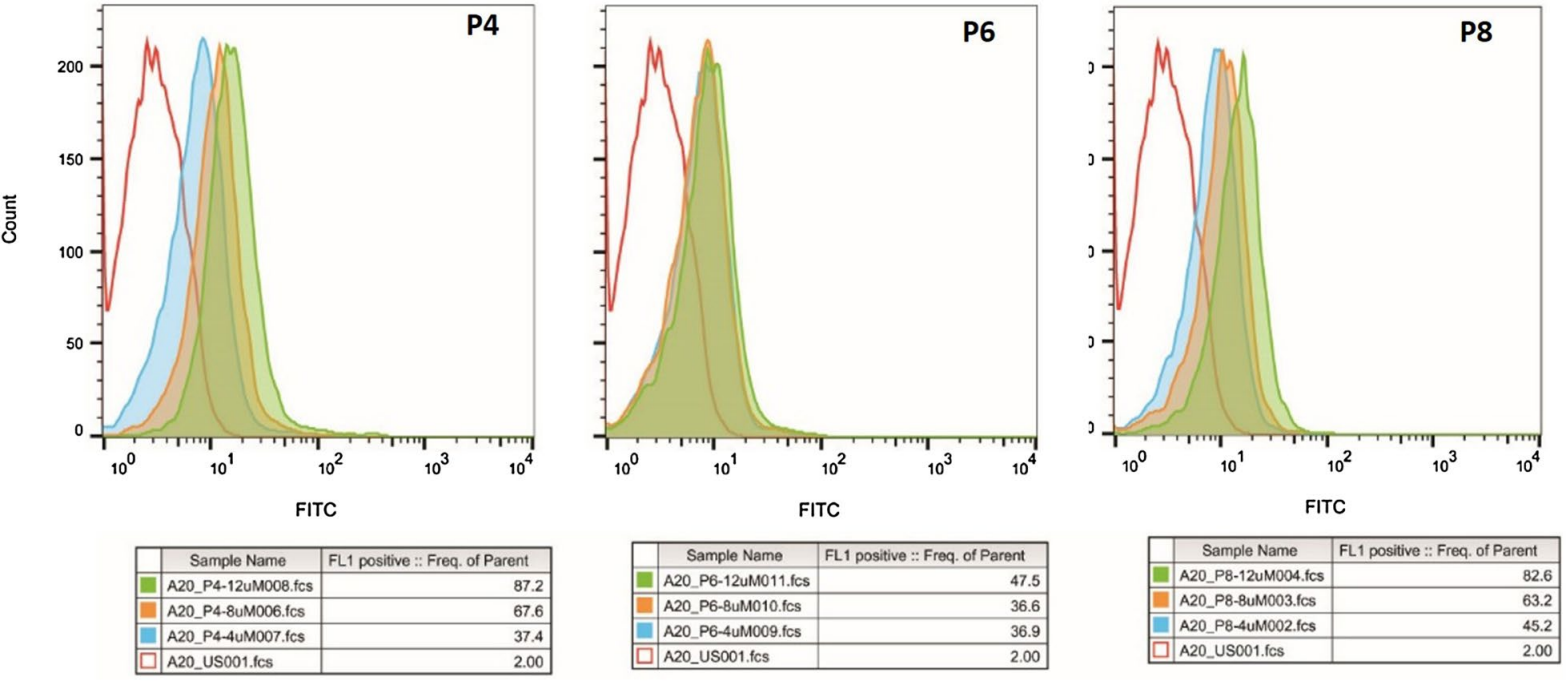
Flow cytometry of the binding potential of Peptide-FITC conjugates for target cells. P4-, P6- and P8-FITC conjugates were incubated at 0, 4 or 8 µM with 10^6^ A20 cells and analyzed for peptide binding. P4 and P8 peptides demonstrated a strong dose-dependent binding to the target A20 cells. These peptides were then tested for binding specificity by exposure to a series of off-target cells as shown in Fig. 3

**Fig. 2:**
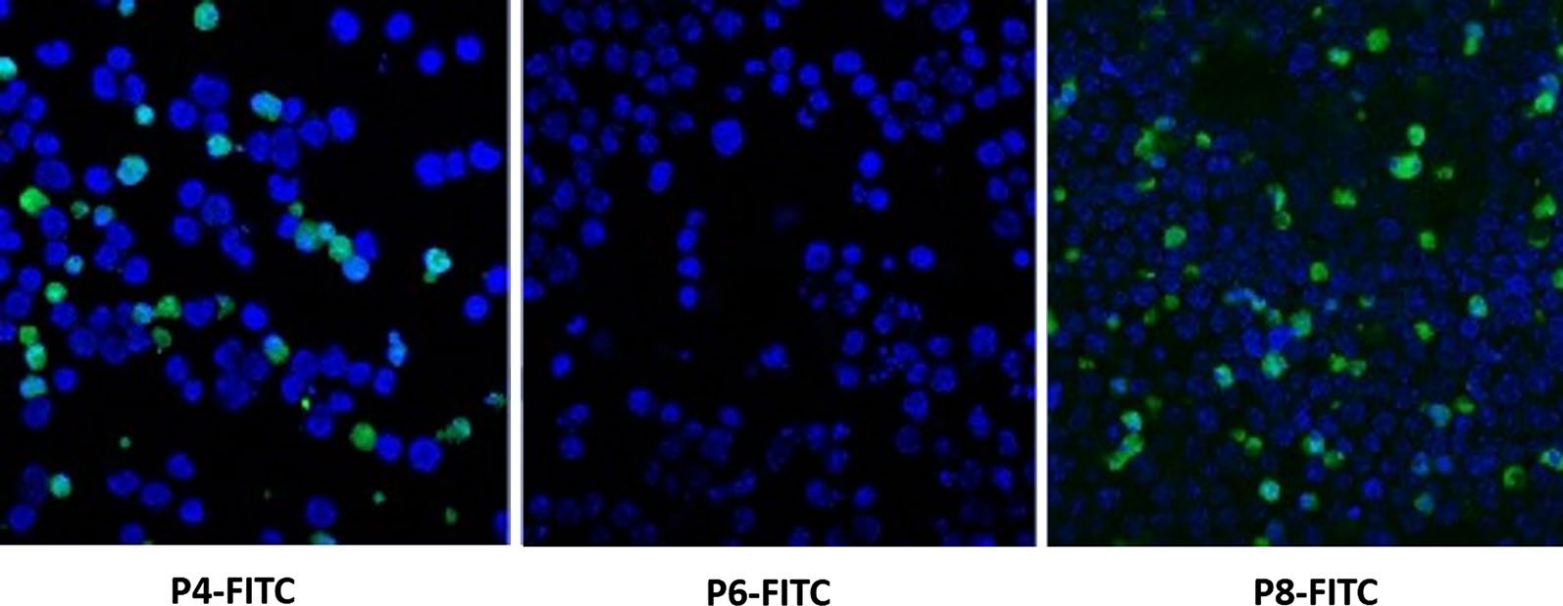
Ability of peptides to internalize into A20 cells. P4, P6 and P8-FITC conjugates were incubated with A20 cells. After several washes and counterstaining with DAPI, the cells were viewed by confocal microscopy. The P6 conjugate did not penetrate into the cells unlike P4 and P8. The FITC marker localization is extra-nuclear

These three peptides were synthesized with or without FITC conjugated to their N-termini. Their binding to target A20 cells was determined by flow cytometry (Fig. 1). The binding of each peptide was dose-dependent, however P4 and P8 bound by almost twice as much as did P6 at most of the concentrations tested. The conjugates were also tested with confocal microscopy to compare peptide internalization into A20 cells as shown in Fig. 2. Clearly, P4 and P8 are taken up by the cells. Surprisingly P6, which also bound to A20 (Fig. 1) did not internalize into the cells.

### Specificity of peptides for target and off-target cells

**Fig. 3:**
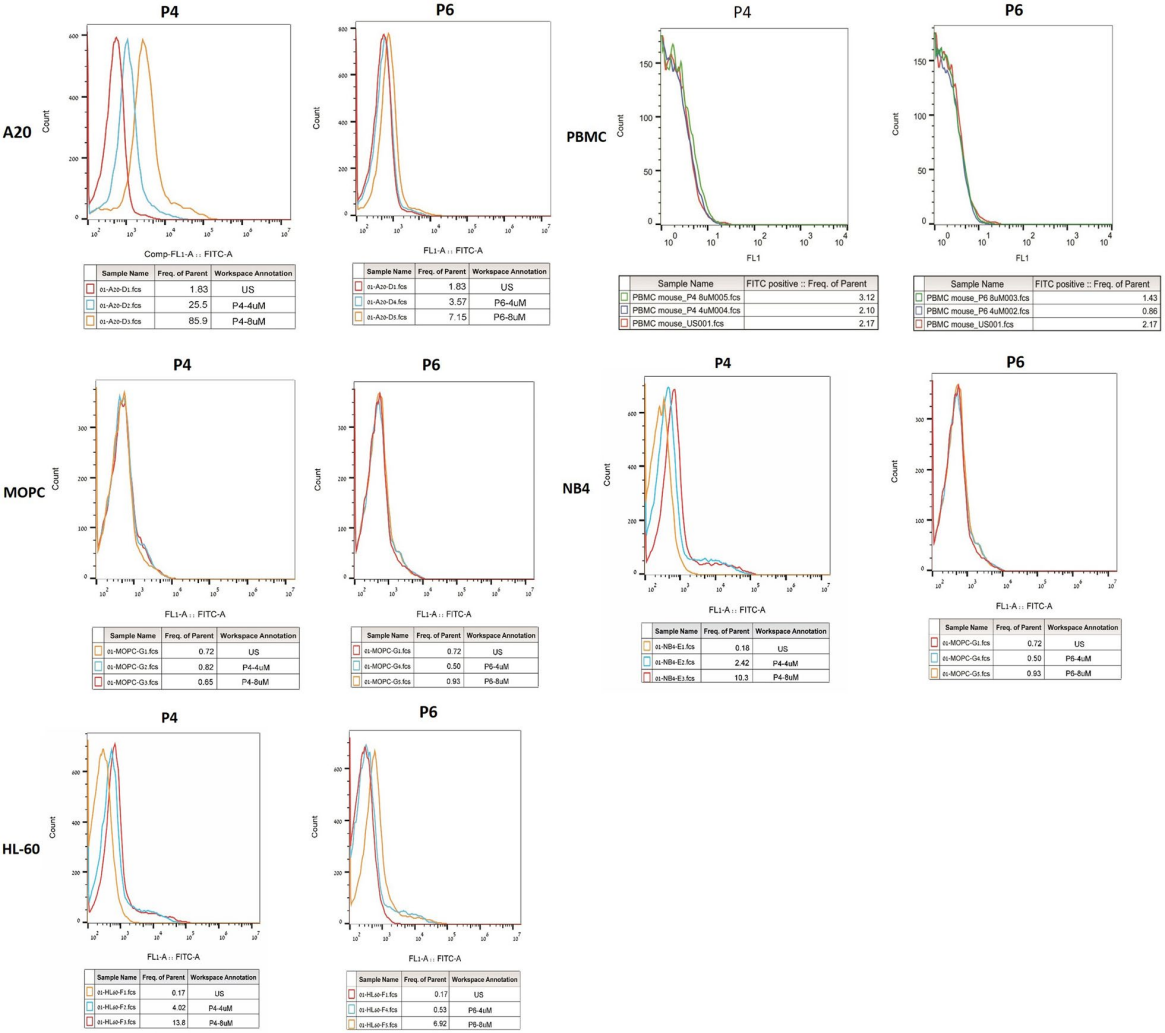
Flow cytometry analysis of P4 and P6 peptides binding to off-target cells. P4- and P6-FITC conjugates were incubated at the indicated concentrations with cells to ascertain the dose-binding response. The data was collected from Becton Dickson FACS Calibur cell analyzer and analyzed with Flow Jo software. Aside from the strong dose–response to A20 target cells P4 binds a small population of NB4 and HL-60 myeloid leukemic cells but not to PBMC or MOPC cells

Based on the results above and the peptide copy numbers from Table 1, we chose to further investigate the activity of P4as a positive binder and P6 as a negative/weak binder. P4-FITCand P6-FITC were tested by flow cytometry for their binding at several concentrations to a panel of off-target lymphoid and myeloid cells, as illustrated in Fig. 3. At 8 µM, neither peptide bound to either human PBMCs or to MOPC 315.BM leukemic cells. P4 bound to a small population of NB4 cells (human acute myeloid leukemia) (10.3%) and HL-60 cells (human acute promyelocytic leukemia) (13.8%), while P6 was negative for NB4 and bound only 6.9% of HL-60 cells. These results suggest that P4 may bind a cell surface component shared with a small sub-population of human myeloid leukemic cells.

### Cytotoxicity of free and peptide conjugated drugs

**Fig. 4:**
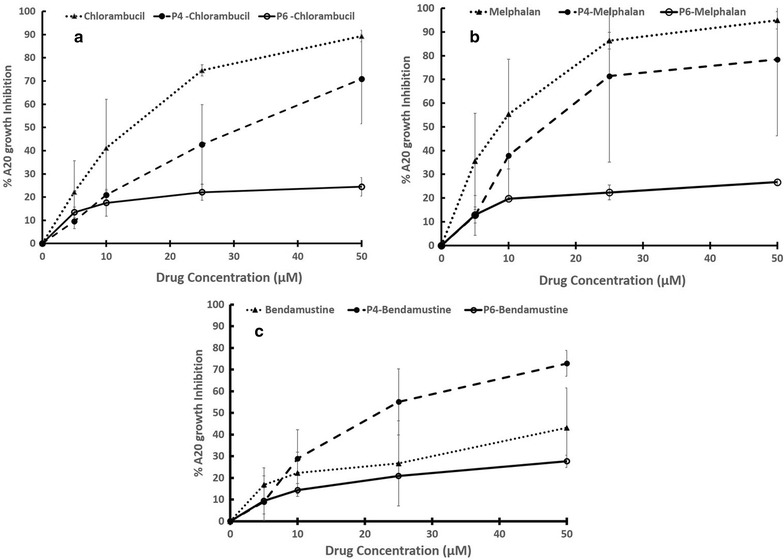
Cell growth Inhibition assay by free and conjugated drugs. Growth inhibiting effect of drug-conjugates VS free drugs was studied on **a** chlorambucil, **b** melphalan, **c** bendamustine. At the end of 72 h incubation period cell growth was assessed using the XTT assay: optical density (OD) was measured at 480 and 680 nm—the latter is the background absorbance. The difference between the 480 and 680 nm measurement was used to calculate the % growth inhibition (GI) in test wells compared with control cells exposed to medium alone. The results shown for each concentration point represent the mean ± standard error for two independent experiments each conducted in (n = 3)

Cells were seeded overnight on microplate culture wells, washed, and re-cultured with fresh medium containing increasing doses (0–50 µM) of free chlorambucil, melphalan or bendamustine or P4- or P6-conjugates of these drugs. After a further 72 h culture, the cytotoxicity of the compounds was determined by measuring cellular metabolism (Fig. 4). The results were expressed as % Growth Inhibition compared to cells not exposed to drug. Clearly A20 cells are significantly more sensitive to Chlorambucil and Melphalan than they are to bendamustine (IC_50_ values 15.8, 9.6, > 50 µM respectively; maximal %GI 89.4, 94.9 and 43.1 respectively). Conjugation of the drugs to P4 affected their efficacy toward A20 cells. For chlorambucil and melphalan, conjugation reduced the cytotoxic effect and this was significant for chlorambucil at 25 µM (p = 0.0013). On the other hand, conjugation significantly improved the cytotoxic effect of bendamustine at 25 (p = 0.043) and 50 µM (p = 0.048). The efficacies of all P6-conjugates were significantly lower than those of P4–conjugates at concentrations above 10 µM.

The spectrum of cytotoxicity of free and P4-conjugated drugs was also tested against three off-target myeloid and lymphoid cell lines. As shown in Additional file 1: Figure S3, the free drugs were equally cytotoxic to all three cell lines. However all the P4-PDCs showed very low activity against these cells, reflecting the FACS binding results in Fig. 3. These results for P4-PDCs in Fig. 4 as well as the P6-PDCs in Additional file 1: Figure S3 show that the conjugates essentially reached a plateau of activity at about 10 µM (drug), demonstrating that delivery of even a 5× excess PDC does not lead to increased uptake of conjugate by off-target cells.

### Stability of P4-drug PDCs

**Fig. 5:**
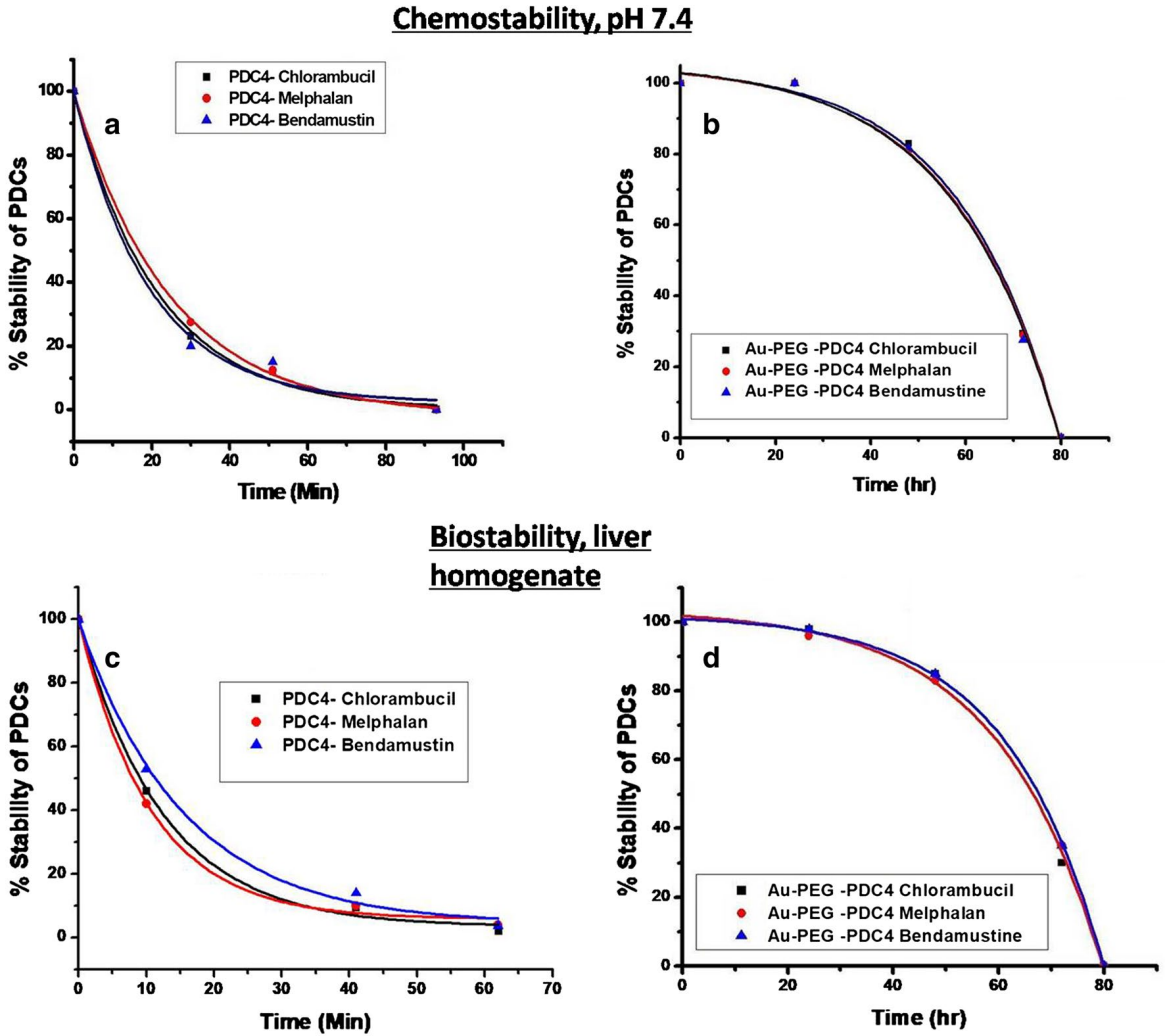
Stability of free PDCs and PDC-PEG-AuNPs. Chemostability was assessed by incubation the constructs in 0.01 M phosphate buffer solution pH 7.2 at 37 °C for different time intervals. Biostability was assessed by incubation in a mouse liver homogenate at 37 °C. At the indicated time intervals, aliquots were taken, filtered or centrifuged (respectively) and analyzed by LC–MS for PDC integrity. Graphs **a** and **c** represent results for PDCs while graphs **b** and **d** represents results for Au-PEG-PDCs. Results are expressed as mean % PDC integrity ± standard error, calculated from two independent experiments

The chemo-stability of the P4-PDCs was tested at a physiological pH of 7.4, while the solutions were incubated at 37 °C. Biostability was assessed after incubation in a mouse liver homogenate. In each case aliquots were taken at selected times and analyzed by LC–MS. PDC integrity was determined by calculating the area under the curve of the PDC peak. The R^2^ values of the exponential decay curves ranged from 0.97 to 0.99 for the three PDCs; the functions of these curves were used to calculate the t½ values. The results (Fig. 5a) showed that after 30 min incubation in buffer, 80% of PDCs had decomposed and they had completely broken down by 1.5 h. The t½ values ranged from 19.3 to 24.6 min (Additional file 1: Table S1 I). In liver homogenate (Fig. 5c), stability decayed more rapidly with half-lives ranging from 10.6 to 15.4 min. There was no significant difference in behavior between the three PDCs and no free drug was observed.

Based on these results, we sought to improve the stability of the PDCs by conjugated them to gold nanoparticles. The physico-chemical parameters of the constructs were characterized and then their biological activity was tested.

### Characterization of PDC-coated PEG-AuNPs

#### Electrophoresis mobility assay

Electrophoretic migration is dependent on the charge and size of dispersed colloidal particles and was used here as a measure of nanoparticle positive charge as a result of peptide co-functionalization. As uncoated AuNPs easily aggregate in TAE electrophoresis buffer, coating with PEG-6000 should allow their dispersion and mobility within the gel, as was indeed the case (Additional file 1: Figure S4, Lane 1). Coating the PEG-AuNPs with PDCs retarded this mobility, indicating a reduction in the overall negative charge of the nanoparticles to slightly different degrees depending on the electrochemistry of both the amino acid sequence and the drug.

#### Size and morphology of the PDC-PEG coated-Au nanoparticles

Coated gold nanoparticles were obtained through a non-covalent coating process. UV–VIS spectra analyses showed no significant change in the 530 nm surface plasmon band peak of the AuNPs as a result of coating them with PEG and PDC (Additional file 1: Figure S5), indicating that conjugation did not significantly change the size of the particles or aggregate them. Additional file 1: Figure S6 depicts the DLS data for these particles.

**Fig. 6:**
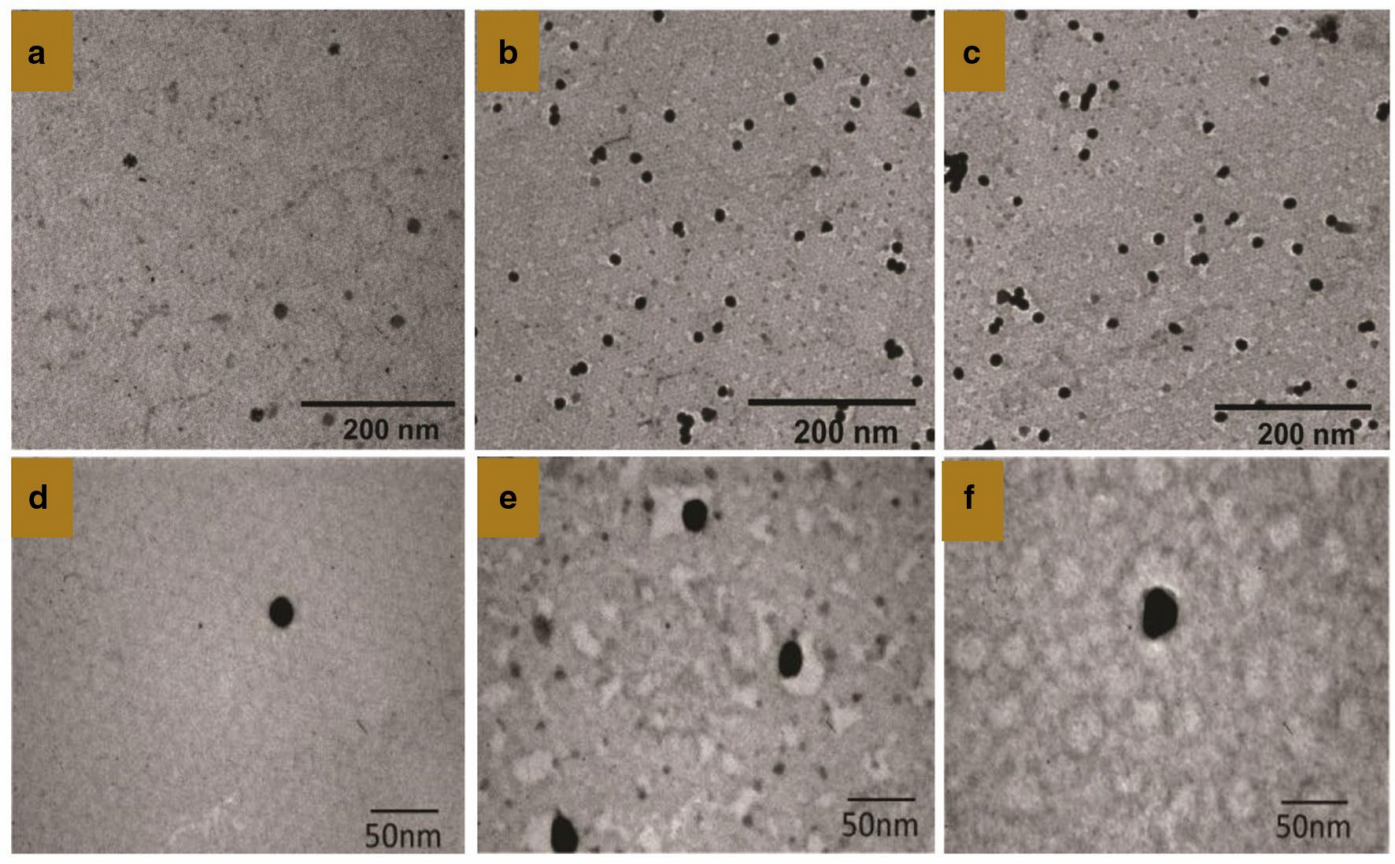
TEM images of PEG-coated gold nanoparticles. Samples were negative stained with 1% phosphotungstic acid; thereafter, images were taken. Scale bars = 200, 20 nm. **a**, **d** Naked Gold nanoparticles (negative stained with 1% phosphotungstic acid; there is no white coated around the nanoparticles (uncoated PEG6000). **b**, **e** Gold nanoparticles coated PEG-6000 (negative stained with 1% phosphotungstic acid) black color gold nanoparticles—white color PEG6000 (negative stain). **c**, **f** Gold nanoparticles coated PEG-6000 + PDC4 − chlorambucil (negative stained with 1% phosphotungstic acid)

Morphology was determined by TEM are shown in Fig. 6. The nanoparticles were spherical in shape with a smooth surface (a and d). After coating them with PEG a characteristic “PEG corona” can be seen (b and e) as has been reported by others [22–24]. There was no significant difference in this morphology when the PEG-AuNPs were coated with PDCs containing P4 and Chlorambucil (c and f), melphalan or bendamustine (data not shown). Dynamic light scattering measurements showed a particle size distribution of 25–40 nm (data not shown).

#### FTIR analyses of coated nanoparticles

**Fig. 7:**
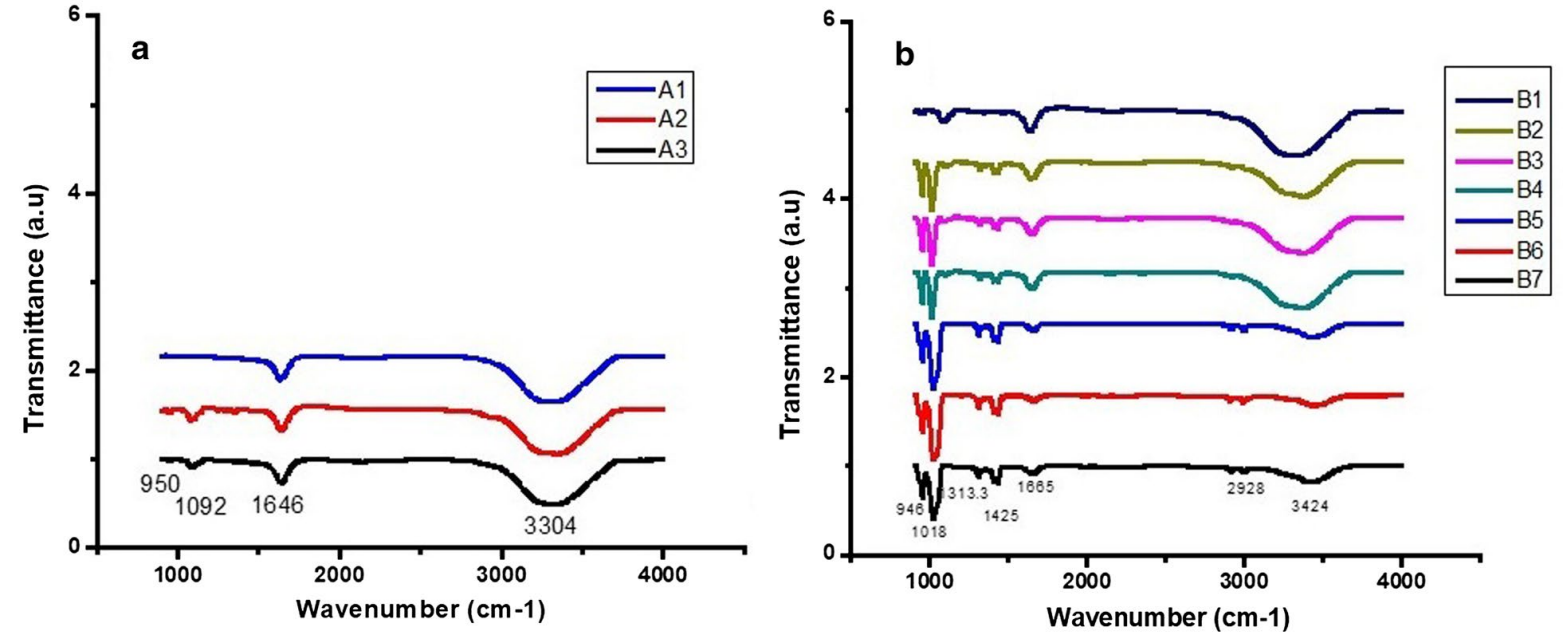
Fourier Transformed Infra-Red spectra of PEG and PDC binding to gold nanoparticles. FTIR analysis was used to characterize the chemical binding of both PEG and Peptide drug conjugate to the surface of the nanoparticle. For the chemical bond significant of each peak, please refer to the text. **a** A1—gold nanoparticles; A2—PEG 6000; A3—PEG-6000 coated gold nanoparticles. **b** B1—PEG-6000 coated gold nanoparticles; B2—gold nanoparticles coated peptide4—chlorambucil; B3—PEG-6000 coated gold nanoparticles—PDC peptide4—melphalan; B4—PEG-6000 coated gold nanoparticles—PDC peptide4—Bendamustine; B5—PDC peptide4—chlorambucil; B6—PDC peptide4– melphalan; B7—PDC peptide4—bendamustine

The nanoparticle conjugates were characterized by FTIR to confirm the chemical binding of both PEG and PDC binding to the surface of the gold nanoparticle. The results are shown in Fig. 7. Both naked and PEG-coated AuNPs displayed signals at 950, 1092, 1646 and 3304 cm^−1^. The presence of hydroxyl groups utilized to link PEG to the oxide surface (Fig. 7a) was confirmed by the absorbance peak for O–H stretch at 1092 cm^−1^ on all nanoparticle spectra. Previous studies showed that nanoparticles functionalized with PEG-NH_2_ display a series of IR absorbance peaks in the range 3300–700 cm^−1^ corresponding to the stretching and bending of several types of chemical bonds [25–28]. PEG-AuNPs coated with P4 PDCs (Fig. 7b) showed additional absorption peaks at, 1425, 1313.3, 1018 and 946 cm^−1^ providing evidence of PDC attachment to the PEG-AuNP.

### Stability of PDC coated PEG-AuNPs

The stability of the PDC-PEG-AuNPs is a key factor for the potential use of AuNPs in targeted drug delivery systems. Initial investigation of its stability in buffer solution at physiological pH 7.4 was carried out. Aliquots were taken at various time points, and the concentrations of released drug were determined by quantitative LC–MS analyses. Figure 5b shows that the PDCs were completely stable over the first 24 h in contrast to the limited stability of PDCs alone (Fig. 5a). After 48 h stability had been reduced by 17% and by 100% after 80 h. The t½ values ranged from 21.0 to 22.3 h. There was no significant difference in behavior between the three PDC conjugates.

### Cytotoxicity of PDC-coated PEG-AuNPs

**Fig. 8:**
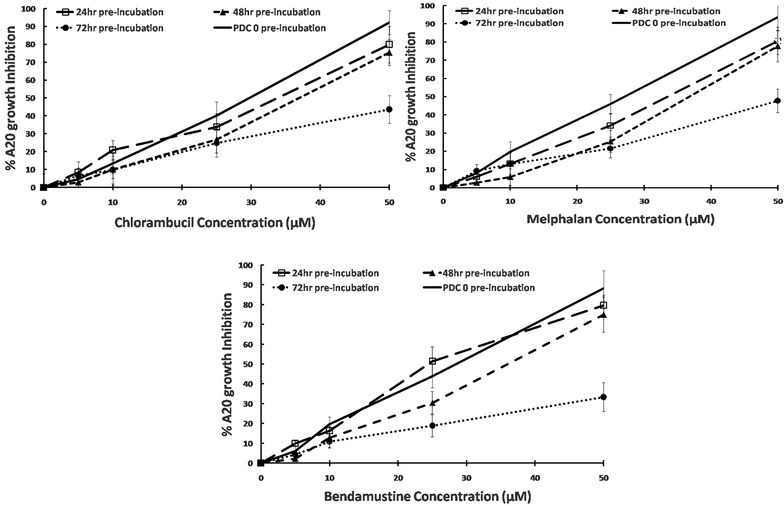
Effect of drug or P4-PDC pre-incubation of cytotoxic activity. Free drugs, free P4-PDCs or P4-PDC-PEG-AuNPs were incubated for 24,48 or 72 h at 37 °C and then added to A20 cells for a further 72 h after which cell growth was assessed using the XTT assay: optical density (OD) was measured at 480 and 680 nm—the latter is the background absorbance. The difference between the 480 and 680 nm measurement was used to calculate the % growth inhibition (GI) in test wells compared with control cells exposed to medium alone. The results shown for each concentration point represent the mean ± standard error for two independent experiments each conducted in (n = 3). Only the results for fresh P4-PDC versus pre-incubated P4-PDC-PEG-AuNPs are shown as pre-incubation of free drugs or free PDC4 abolished their cytotoxic activity by more than 93%

We then tested whether the enhanced stability of the AuNP conjugated P4-PDC could be garnered to extend the cytotoxic activity of the P4-PDC. Free drugs, free PDC4 and P4-PDC-PEG-AuNPs were pre-incubated in culture medium for 24, 48 or 72 h at 37 °C and then added to fresh A20 cells for a further 72 h. Figure 8 shows the results, corrected for cytotoxicity induced by gold particles alone (5–12% from lowest to highest concentration). All three P4-PDC-coated gold nanoparticles pre-incubated for 24 or 48 h induced statistically similar cytotoxicity in A20 to that induced by freshly prepared PDC4 and to coated particles without pre-incubation (the latter data not shown). After 72 h pre-incubation, cytotoxic activity of all three P4-PDC was significantly lower than fresh P4-PDC at the highest concentration (p < 0.01). In contrast, pre-incubation of free drug or free P4-PDC, even for 24 h reduced their cytotoxic effect by > 93% (data not shown). The target cell specificity of the P4-PDC-coated nanoparticles did not react against several off-target cells (Additional file 1: Figure S9).

## Discussion

One of the main goals of targeted drug delivery systems is to reduce unwanted toxicities by delivering the therapeutic war-head specifically to pathologic cells, thereby reducing the exposure of healthy cells to the drug. In this regard phage display peptide technology is a powerful tool for the discovery of novel peptides that can be used to target cytotoxic drugs to cancer cells [29]. The flexible use of these peptides to generate mono- and dual drug peptide-drug-conjugates [7, 8, 21] is offering new possibilities to develop more effective cancer therapies [30].

Nonetheless, the stock phage pool contains many clones with low affinity for candidate target receptors. These clones might lead to a cross-reactive or non-specific binding overload that successfully competes with less abundant, but higher affinity clones. Therefore prior to exposing the stock phage library to our A20 target leukemic cells, we removed from the library clones with affinity for cell surface components present on several types of off-target cells. We surmised that this strategy might increase the possibility of fishing out clones that bind unique receptor(s) on the A20 target cells. Indeed the procedure significantly reduced the library size almost 5 order of magnitude (Additional file 1: Figure S2).

Initially, the goal of our study was to monitor the pharmacokinetics and anti-cancer efficacy of novel lymphoma PDCs using the A20 cell line as a model. Using the “absorbed phage library”, we discovered several clones internalized by the target cells (Table 1). We chose to study P4, P6 and P8. The P4 clone was repeated a number of times amongst those clones sequenced and may have higher affinity for a receptor. P2 was overlooked because its predicted net positive charge of 0.8 at pH 7.4 (data not shown) may lead to non-specific electrostatic binding to cell surface components. On the other hand P6 and P8 are predicted to have negative charges at pH 7.4 (− 2.2 and − 1.2 respectively) due to the presence of carboxylate moiety of aspartic acid. P4, P6 and P8 were synthesized and shown to bind the target cells (Fig. 1), but only P4 and P8 actually internalized into the cells in their free form (Fig. 2). This result underscores the importance of verifying that a displayed candidate peptide behaves similarly in the free form to that displayed as an appendage to an exposed phage protein.

The suitability of P4 as a candidate drug carrier for our model was strengthened by flow cytometry studies showing that while A20 is of B-cell origin, the peptide did not bind to either to a murine myeloma B cell line or to normal human lymphocytes (Fig. 3). It did however appear to bind a small population of human myeloid derived leukemic cells. We tested the cytotoxic efficacy of three chemotherapeutics in their free form and after conjugation to P4. Figure 4 shows that A20 cells were less sensitive to bendamustine than to chlorambucil or melphalan. Arimany-Nardi et al. [31] recently reported that Bendamustine enters the cell through the hOCT1 transporter which is expressed on some lymphoma cells [32] although the protein’s expression on A20 cells is not known. In contrast, early studies showed that Chlorambucil is taken up by simple diffusion [33, 34**]**, however it too is not taken up by all leukemic cells [4, 6]. Melphalan is actively taken up by L-type amino acid transporter 1 (LAT1) which is over-expressed in many types of cancer cells [35, 36].

Incorporation of the drugs into PDCs reduced the dose–response effect of Chlorambucil and Melphalan but significantly improved the effect of Bendamustine. These variations are to be expected given that PDC incorporation will depend on the receptor cell surface density and the mechanism of internalization. Indeed conjugation to P4 resulted in their being no significant difference in the dose–response curves of the three PDCs even at the 25 µM dose. Interestingly, bendamustine has been reported to be more effective clinically against B-cell leukemias [37, 38], and to possess additional anti-cancer mechanisms of action [39] than chlorambucil. Our results indicate that at least against the A20 leukemic-like cell line the two drugs are equally effective when delivered as PDCs.

PDC stability was assessed in buffer at physiological pH and after incubation in mouse liver homogenate. In buffer, the t½ values ranged from 19.3 to 24.6 min while in liver homogenate they ranged from 10.6 to 15.4 min (Fig. 5a). By assessing the molecular mass of new LC–MS peaks appearing during the incubation period, we determined that the loss of PDC integrity was mostly due to the hydrolysis of the two chlorines on all three nitrogen mustard drugs, a process which was earlier reported to lead to loss of alkylating activity [40].

To improve the stability of the PDCs, we attached them to PEG-coated gold nanoparticles and characterized the physico-chemical parameters of the resulting conjugates (Additional file 1: Figures S3, S4 and Figs. 6, 7). While many types of nanoparticles have been tested as potential vehicles for targeted cancer drug delivery [11, 13], the idea of using gold particles as a mechanism to stabilize PDCs and thus extend their bioavailability has not yet been reported. Indeed this strategy significantly extended the chemical and biological half-lives of the PDCs to 21.0–22.3 h (Fig. 5b). Conjugation to AuNPs also opens up the possibility for developing prolonged targeted cancer cell treatment protocols. We suggest this because pre-incubation of free drugs or free PDCs for 24 h prior to their exposure to cells reduced their ability to kill A20 targets by > 90% (data not shown). By contrast, even after 48 h pre-incubation, the PDC4-PEG-AuNPs retained their cytotoxic capacity (Fig. 8).

Our data suggest a new avenue of application for gold nanoparticles in oncology, including hematological cancers [12], by directly combining them with the potential for targeted cancer therapy of PDCs [21, 30, 41]. The ability of AuNPs to extend the bioavailability of PDCs may circumvent the need for the particles to actually penetrate the tumor, a noted limitation with some nanoparticles [18]. This strategy may lead to slow release, targeted drug delivery systems which might significantly benefit cancer patients undergoing chemotherapy.

## Conclusions

Peptide-drug-conjugates have demonstrated a number of chemical, structural and biological advantages that hold potential for improving the targeted delivery and efficacy of chemotherapeutic drugs. However one limitation to their clinical application is the relatively short half-live of the constructs in biological fluids and tissue. Our results suggest that this hurdle can be overcome by easily conjugating them to pegylated-gold nanoparticles. By significantly extending PDC stability, this conjugation also opens up the possibility of developing slow release formulations of targeted drug delivery systems containing PDCs.

## Additional file

**Additional file 1.** Additional figures S1–S9 and table S1.

## Abbreviations

TDD: targeted drug delivery
PDC: peptide drug conjugate
AuNP: gold nanoparticles
PEG: polyethylene glycol
FITC: fluorescein thiocyanate
PBMC: peripheral blood mononuclear cells
LC–-MS: liquid chromatography–mass spectroscopy
TAE: Tris-acetic acid-EDTA buffer
FTIR: Fourier transformed infrared spectroscopy
