# Supplementary material for: Gold nanoparticles stabilize peptide-drug-conjugates for sustained targeted drug delivery to cancer cells
Source: J Nanobiotechnology. 2018 Mar 30;16:34. doi: 10.1186/s12951-018-0362-1 (PMC5877385; doi:10.1186/s12951-018-0362-1)
Supplement: Supplementary file 1 — Additional file 1. Additional figures S1–S9 and table S1. [file 12951_2018_362_MOESM1_ESM.docx]

**Gold nanoparticles stabilize peptide-drug-conjugates for sustained targeted drug delivery to cancer cells**

**Kalimuthu et al. Additional file 1**

**
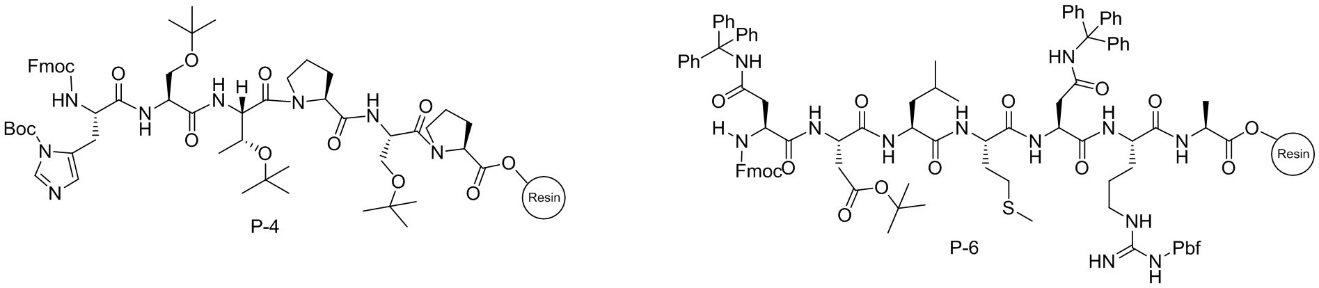
**

**Figure S1.** Structures of P4 and P6 peptides bound to 2-chlorotrityl resin


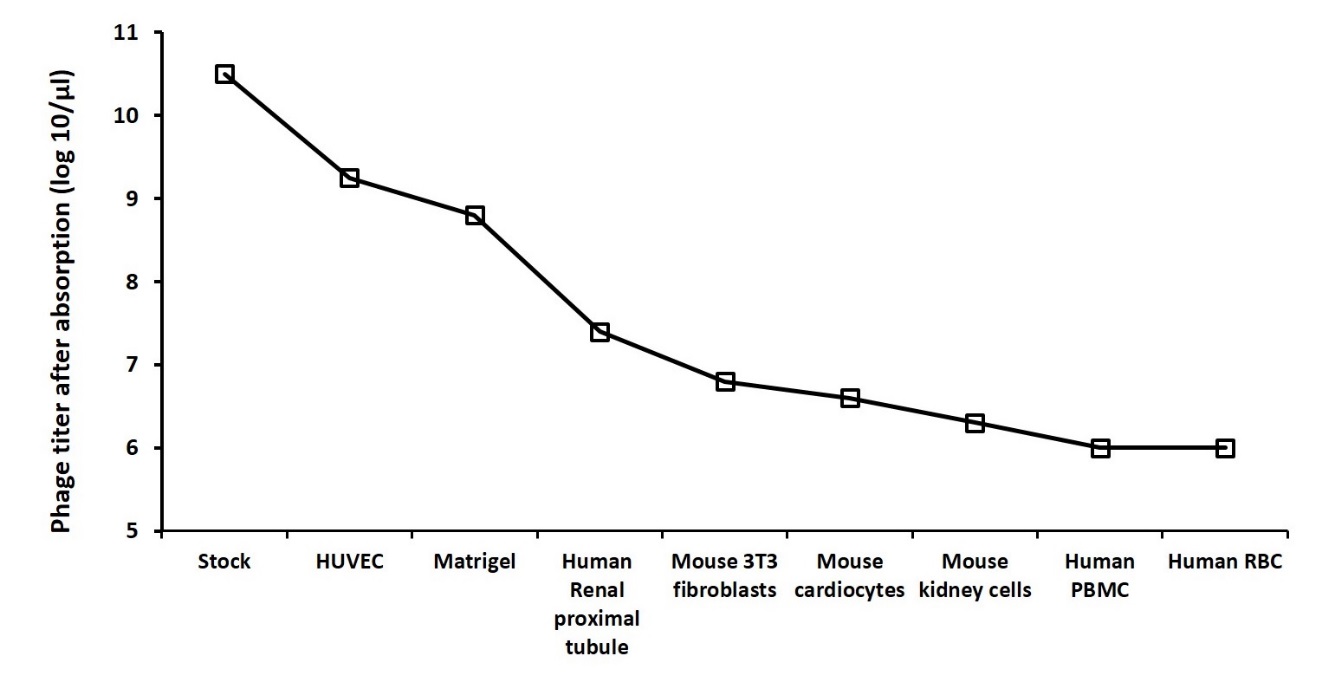


**Figure S2:**Decrease in Log_10_ phage titer following sequential in vitro absorption of stock Ph.D 7 linear phage display library on a series of normal human and mouse cells and on matrigel. In each case, 1x10^6^log-phase cells were washed with PBS and re-suspended in 1ml of RPMI. 10 microliters of phage particles (2X10^11^) were added 1 ml of cells and the mixture incubated for 1h at 37^0^C with gentle shaking. The mixture was then centrifuged at 1500 rpm and the supernatant containing the unbounded phage was collected. A small portion (3-5µl) was retained and titrated to determine the decrease in phage titer resulting from each absorption. The remainder was used for the next stage of negative panning. The library was similarly exposed to matrigel.


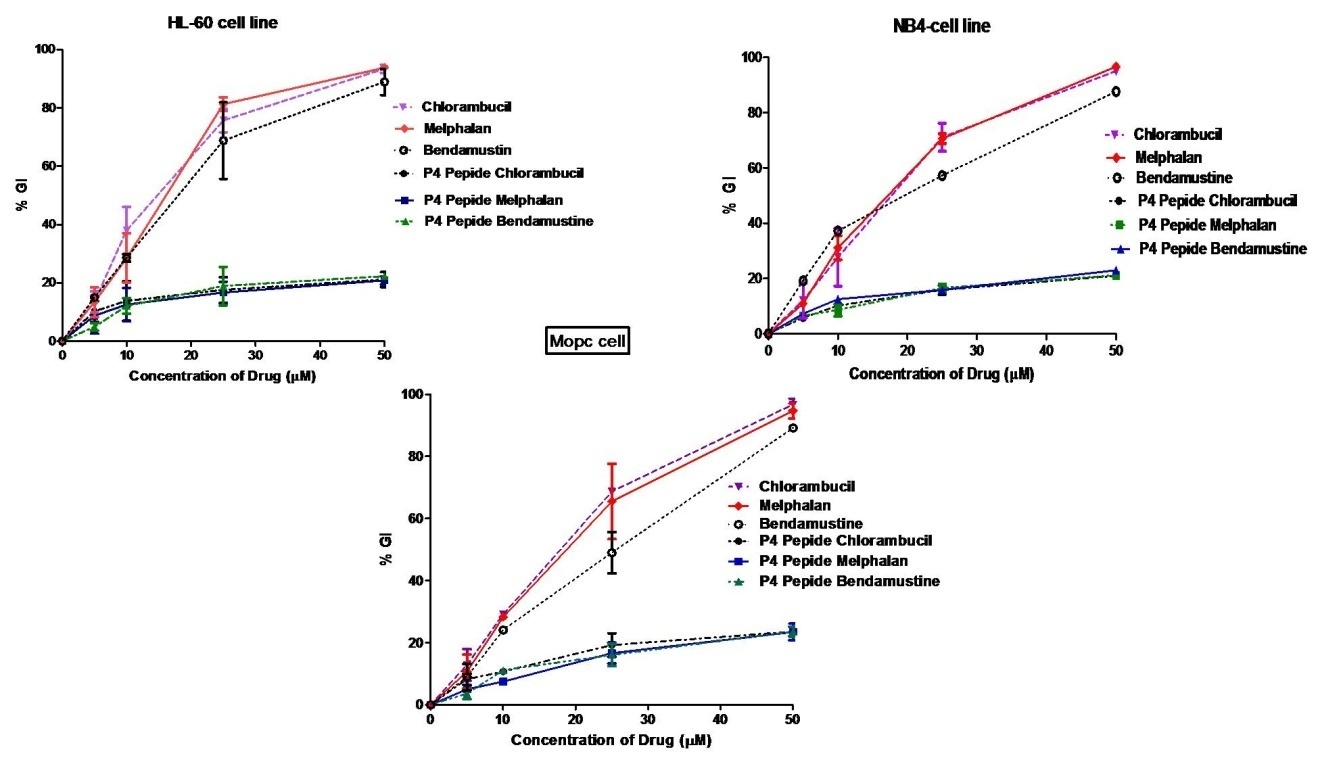


**Figure S3:** Cytotoxic effect of free drugs of P4-PDCs against off-target cells. The compounds were cultured with cells for 72 hrs after which cell growth was assessed using the XTT assay: Optical density (OD) was measured at 480 and 680 nm – the latter is the background absorbance. The difference between the 480 and 680 nm measurement was used to calculate the % growth inhibition (GI) in test wells compared with control cells exposed to medium alone. The results shown for each concentration point represent the mean ± standard error for independent experiments each conducted in triplicate


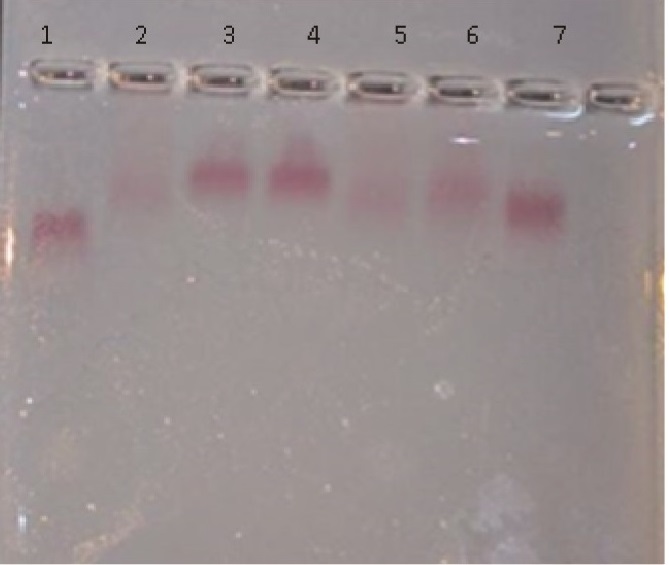


**Figure S4:** Electrophoretic mobility of AuNPs and PDC-AuNPs. Samples were electrophoresed in a 1.5% agarose gel in TAE running buffer. **Lane 1**: PEG-6000 AuNP; **Lane 2**: PEG-6000 AuNP-P4–Chlorambucil; **Lane 3**: PEG-6000 AuNP-P4–Melphalan; **Lane 4**: PEG-6000 AuNP P4-Bendamustine; **Lane 5**: PEG-6000 AuNP P6-Chlorambucil; **Lane 6**: PEG-6000 AuNP P6-Melphalan; **Lane 7**: PEG-6000 AuNP P6-Bendamustine


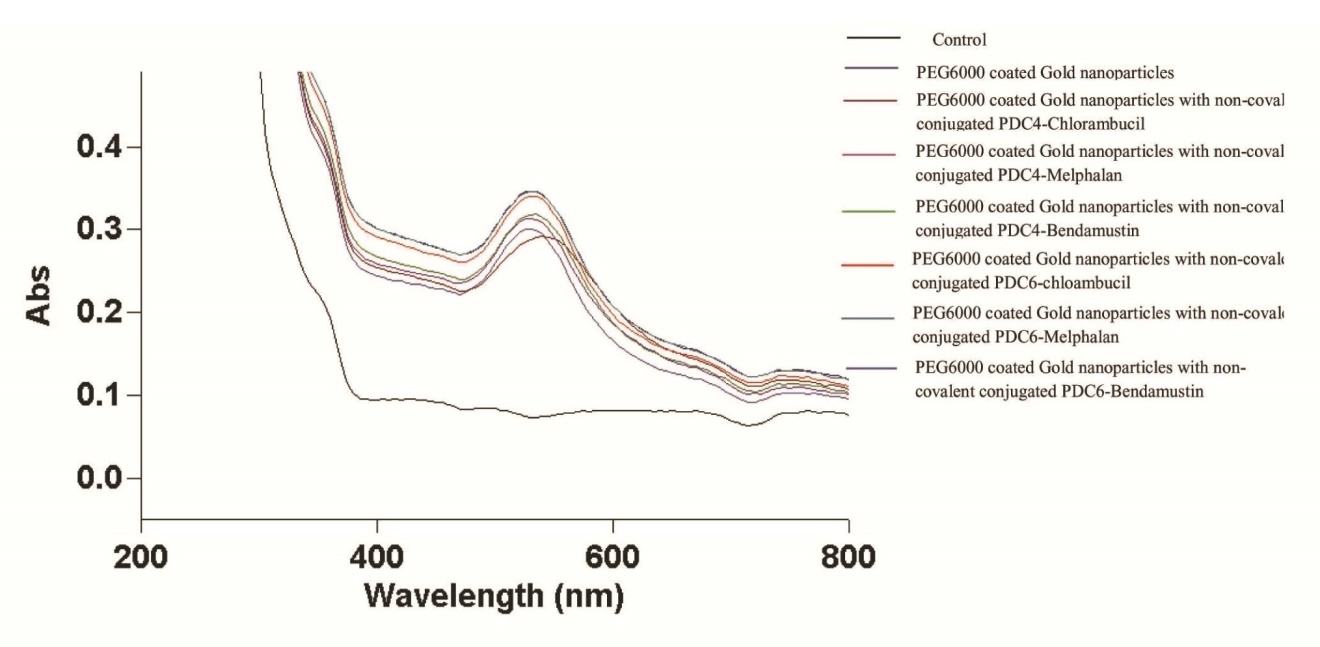


**Figure S5**

UV-VIS spectra in the range of 100-800nm of PEG-coated gold particles along and those non-covalently coated with peptide-drug-conjugates (PDCs). The PDCs were composed of either P4 or P6 peptides conjugated with wither Chlorambucil, Melphalan or Bendastumin. The major peak at 530nm showing PEG conjugation to the particles was not affected by additional attachment of the PDCs.


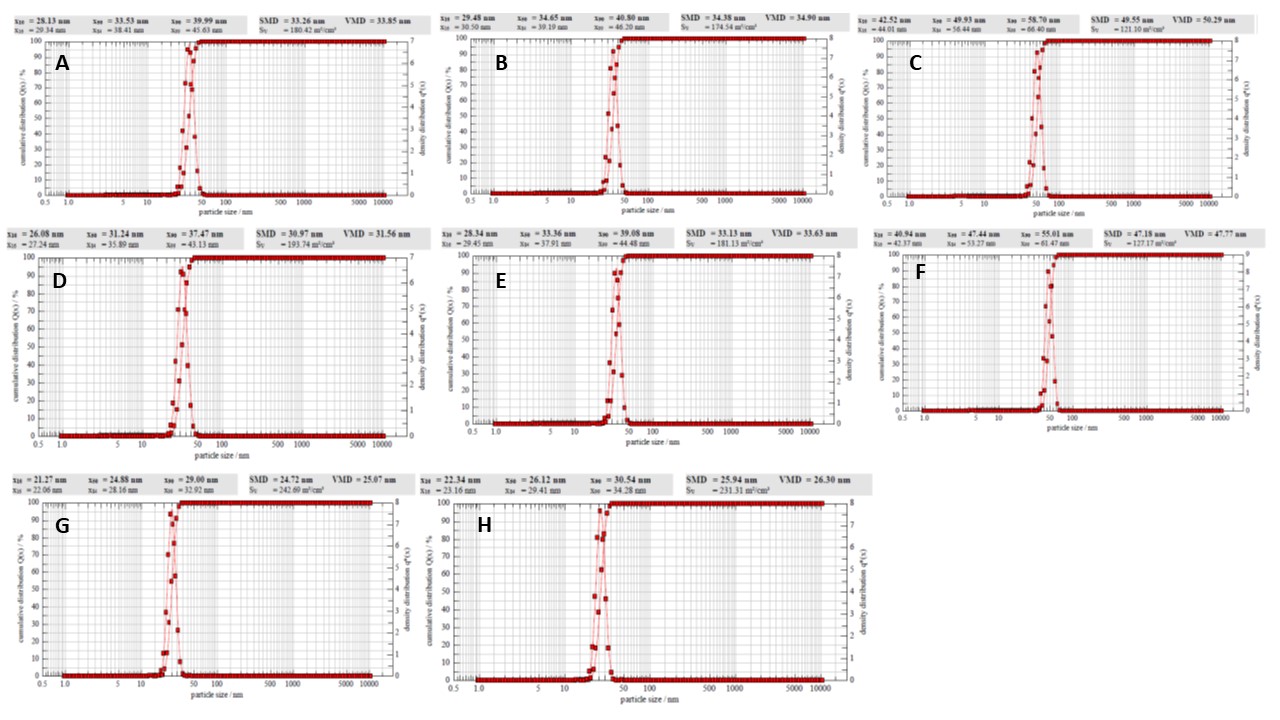


**Figure** **S6** DLS size characterization of the PDC-PEG-AuNP

**(A**) PEG-6000 AuNP-P4–Chlorambucil;(**B**) PEG-6000 AuNP-P4–Melphalan; **(C**) PEG-6000 AuNP P4-Bendamustine; **(D**) PEG-6000 AuNP P6-Chlorambucil; (**E**) PEG-6000 AuNP P6-Melphalan; (**F**) PEG-6000 AuNP P6-Bendamustine**;** (H) AuNP**; (G**)PEG-6000 AuNP.

**A**

**B**

**C**

**D**

**E**

**F**

**Figure** **S7.** Elution time and Mass spectroscopy of P4 PDCs

**(A**) P4–Chlorambucil- *Elution time* (**B**) P4–Chlorambucil –Mass spectra **(C**) P4–Melphalan-- *Elution time* **(D**) P4–Melphalan- Mass spectra (**E**) P4-Bendamustine- *Elution time* (**F**) P4-Bendamustine- Mass spectra**.**

**A**

**B**

**C**

**D**

**E**

**F**

**Figure** **S8** Elution time and Mass spectroscopy of P6 PDCs

**(A**) P6–Chlorambucil- *Elution time* (**B**) P6–Chlorambucil –Mass spectra **(C**) P6–Melphalan- *Elution time* **(D**) P6–Melphalan- Mass spectra (**E**) P6-Bendamustine- *Elution time* (**F**) P6-Bendamustine- Mass spectra**.**

**
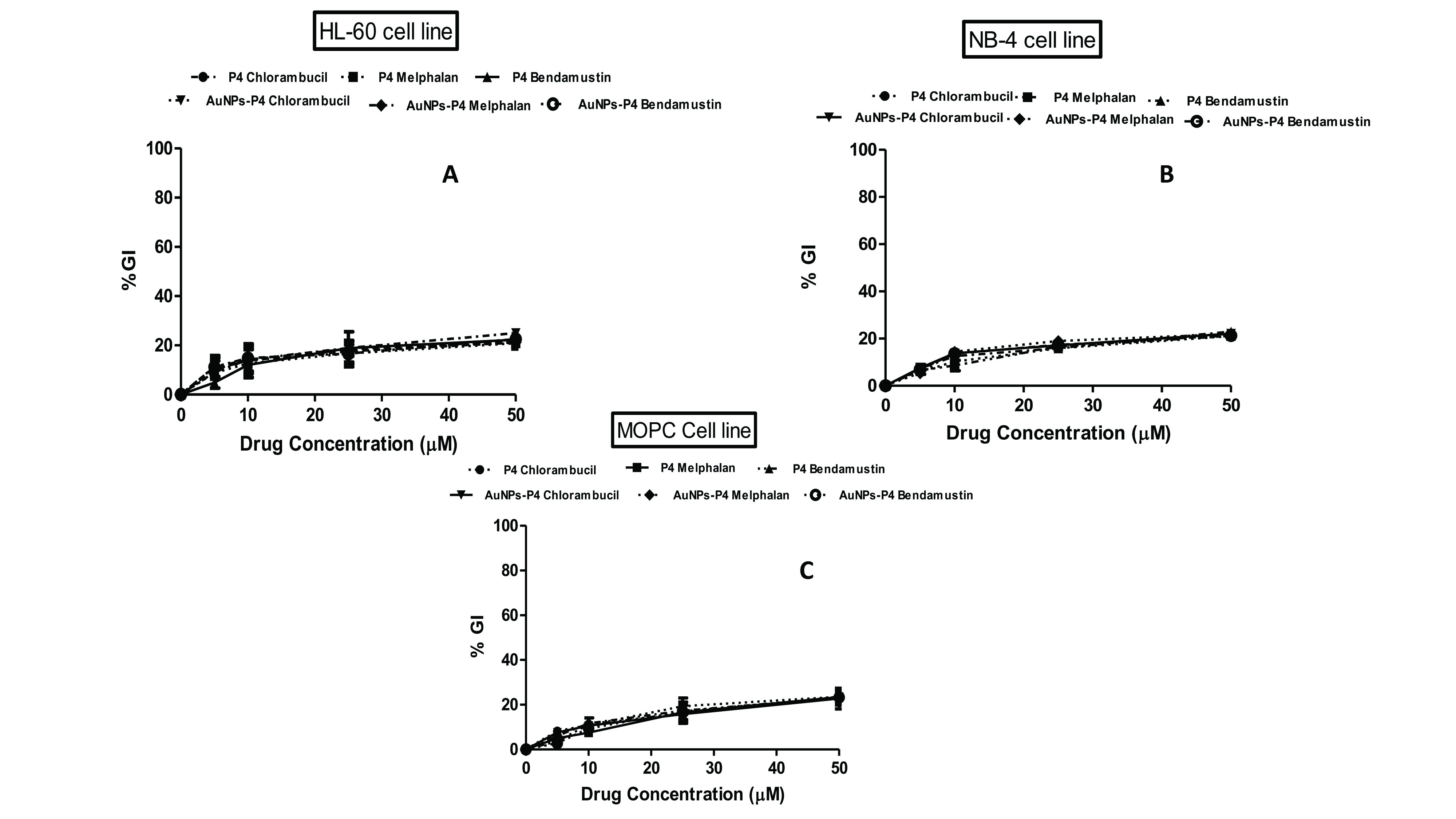
**

**Figure S9** Cell growth Inhibition of Peptide drug conjugates and AuNPs –Peptide drug conjugates on off-target cells. P4-PDC or P4-PDC-PEG-AuNPs were cultured with (A) HL-60 cells ((B) NB-4 cells or (C) MOPC cells. At the end of 72 hr incubation, cell growth was assessed using the XTT assay: Optical density (OD) was measured at 480 and 680 nm – the latter is the background absorbance. The difference between the 480 and 680 nm measurement was used to calculate the % growth inhibition (GI) in test wells compared with control cells exposed to medium alone. The results shown for each concentration point represent the mean ± standard error for two independent experiments each conducted in triplicate.

**Table S1:** Calculated chemo- and bio-stabilityt½ values of PDCs and PDC-PEG-AuNPs

| **Material** | **Chemostability**  **(pH 7.4)** | **Biostability**  **(liver homogenate)** |
| --- | --- | --- |
| **P4-Chlorambucil** | 21.5 mins | 12.4 mins |
| **P4-Melphalan** | 24.6 mins | 10.6 mins |
| **P4-Bendamustine** | 19.3 mins | 15.4 mins |
| **P4-Chlorambucil-PEG-AuNP** | 21.0 hrs | 20.0 hrs |
| **P4-Melphalan-PEG-AuNP** | 22.0 hrs | 18.3 hrs |
| **P4-Bendamustine-PEG-AuNP** | 22.3 hrs | 18.3 hrs |
